# Growth and Photosynthetic Efficiency of Microalgae and Plants with Different Levels of Complexity Exposed to a Simulated M-Dwarf Starlight

**DOI:** 10.3390/life13081641

**Published:** 2023-07-28

**Authors:** Mariano Battistuzzi, Lorenzo Cocola, Elisabetta Liistro, Riccardo Claudi, Luca Poletto, Nicoletta La Rocca

**Affiliations:** 1 National Council of Research of Italy, Institute for Photonics and Nanotechnologies (CNR-IFN), 35131 Padua, Italy; lorenzo.cocola@cnr.it (L.C.);; 2 Department of Biology, University of Padua, 35121 Padua, Italynicoletta.larocca@unipd.it (N.L.R.); 3 Center for Space Studies and Activities (CISAS), University of Padua, 35131 Padua, Italy; 4 National Institute for Astrophysics (INAF), Astronomical Observatory of Padua, 35122 Padua, Italy; 5 Department of Mathematics and Physics, University Roma Tre, 00146 Rome, Italy

**Keywords:** M-dwarf spectrum, laboratory simulations, oxygenic photosynthesis, light acclimation, microalgae, *Physcomitrium patens*, *Arabidopsis thaliana*

## Abstract

Oxygenic photosynthetic organisms (OPOs) are primary producers on Earth and generate surface and atmospheric biosignatures, making them ideal targets to search for life from remote on Earth-like exoplanets orbiting stars different from the Sun, such as M-dwarfs. These stars emit very low light in the visible and most light in the far-red, an issue for OPOs, which mostly utilize visible light to photosynthesize and grow. After successfully testing procaryotic OPOs (cyanobacteria) under a simulated M-dwarf star spectrum (M7, 365–850 nm) generated through a custom-made lamp, we tested several eukaryotic OPOs: microalgae (*Dixoniella giordanoi*, *Microchloropsis gaditana*, *Chromera velia*, *Chlorella vulgaris*), a non-vascular plant (*Physcomitrium patens*), and a vascular plant (*Arabidopsis thaliana*). We assessed their growth and photosynthetic efficiency under three light conditions: M7, solar (SOL) simulated spectra, and far-red light (FR). Microalgae grew similarly in SOL and M7, while the moss *P. patens* showed slower growth in M7 with respect to SOL. *A. thaliana* grew similarly in SOL and M7, showing traits typical of shade-avoidance syndrome. Overall, the synergistic effect of visible and far-red light, also known as the Emerson enhancing effect, could explain the growth in M7 for all organisms. These results lead to reconsidering the possibility and capability of the growth of OPOs and are promising for finding biosignatures on exoplanets orbiting the habitable zone of distant stars.

## 1. Introduction

Prime targets in the search for life beyond the solar system are the exoplanets orbiting in the Habitable Zone (HZ) around M-dwarf stars. In the last couple of decades, more and more exoplanets were discovered thanks to the surveys of space missions such as Kepler and TESS [1,2], with most of the rocky terrestrial-like bodies found orbiting these stars [3,4,5,6]. M-dwarfs are the most abundant stars known in the Milky Way and could theoretically allow life evolution due to their long lives [7]. However, M-dwarf stars have different spectral characteristics with respect to the Sun, being far less luminous and generating a light spectrum with a major component in far red (FR, 700–750) and infrared (IR, 750–1000 nm) while emitting very low in the visible (VIS, 400–700 nm).

This peculiar light spectrum has led researchers to wonder if oxygenic photosynthesis, the most prominent biological process that shaped life evolution on our planet, could work on exoplanets orbiting those stars. VIS light is the region of the electromagnetic spectrum called “photochemically active radiation” (PAR). PAR photons are harvested via chlorophyll *a* (Chl *a*) and other accessory pigments of oxygenic photosynthetic organisms (OPOs) to drive the primary production of organic compounds and release molecular oxygen (O_2_), fundamental molecules that are then made available to all other organisms. OPOs represent almost 99% of the Earth’s biomass [8] and generate atmospheric and surface biosignatures, which makes them ideal targets for investigating the detectability of life beyond Earth [9]. This is linked, respectively, to their oxygen release activity and to the absorption of their photosynthetic pigments, which generate a distinctive Earth reflectance spectrum [9].

The possibility of oxygenic photosynthesis in the HZ of M-dwarfs has been so far vastly discussed using models and a theoretical approach [10,11,12,13,14,15,16,17,18], but, except experimental work on cyanobacteria [19,20], investigations into more complex photosynthetic organisms’ responses to simulated M-dwarfs spectra are missing.

In our previous works, we focused our attention on the most ancient OPOs, cyanobacteria. Cyanobacteria first evolved oxygenic photosynthesis in early Earth, inducing the first oxygenation event of our planet around 2.4–2.1 BYA [21]. Cyanobacteria have high metabolic plasticity and adaptability, which have allowed them to acclimate and adapt to different light intensities and spectra of niches inhospitable for most photosynthetic organisms [22]. On independent places on Earth, a restricted number of cyanobacteria have been shown to be able to utilize FR light alone or in addition to VIS light to carry oxygenic photosynthesis via the so-called Far-Red Light Photoacclimation (FaRLiP) [23,24]. FaRLiP cyanobacteria were deemed ideal model organisms when considering the possibility of oxygenic photosynthesis on exoplanets orbiting M-dwarfs as well as in caves and lava tubes [25], given their lighting environments [20,26]. We, thus, investigated the survival, growth, acclimation capabilities, and photosynthetic capacities of several strains of cyanobacteria exposed to a simulated M-dwarf starlight, demonstrating that cyanobacteria cannot only survive but even efficiently harvest the M-dwarf light spectrum, regardless of their ability to utilize specific FR light acclimation strategies [19,20].

More complex life forms, such as algae and plants that eventually colonized land, have evolved after the first event of the Earth’s atmosphere oxygenation by cyanobacteria. Photosynthetic eukaryotes appeared around ~900 MYA [27] from an endosymbiotic event between a eukaryotic heterotroph and a cyanobacterium [28,29]. The acquisition of photosynthesis by the eukaryotes was a success, and so they spread on Earth rapidly, taking the role of major primary producers, challenging cyanobacteria for ecological niches, and colonizing different environments, both terrestrial and aquatic [30]. Secondary endosymbiotic events later occurred between heterotrophic and chloroplast-containing eukaryotes, further diversifying photosynthetic eukaryotes [30,31]. The global distribution of photosynthetic eukaryotes significantly contributed to the second oxygen rise that peaked during the Carboniferous (~360–300 MYA) [21] and eventually stabilized this atmospheric gas at the ~20% level we see today.

Regardless of their complexity, all OPOs perform oxygenic photosynthesis using the same cyanobacteria complexes, comprising photosystems I and II (PSI and PSII), reaction centers constituted of very conserved pigment–protein complexes embedded in the thylakoids (photosynthetic membranes). The associated light-harvesting antenna systems have, instead, different organizations and compositions depending on the taxa. These complexes have different light absorption capabilities based on the specific interactions between pigments and proteins constituting the antennae; nearly all OPOs use chlorophyll *a* (Chl *a*) as a major pigment together with other accessory pigments, all absorbing the VIS light of the sunlight spectrum.

Also among photosynthetic eukaryotes FR acclimations and adaptations have been observed. Some eukaryotic algae derived from secondary endosymbiosis can harvest FR light by changing the organization of antenna complexes without synthesizing new, FR-absorbing pigments as cyanobacteria do [32]. Under FR light, light-harvesting complexes of these algae can aggregate, changing pigment–protein and pigment–pigment interactions, leading to a red shift in the absorption properties of chl *a*; this results in the absorption of light above 700 nm, as seen in their in vivo absorption spectra [33,34,35].

Land plants instead, especially heliophyte (Sun-adapted) higher plants, are not particularly efficient at absorbing FR light alone, and only sciophyte (shade-adapted) can grow under spectra characterized by low VIS associated with FR-enriched light [36] since they can absorb longer wavelengths through the so-called red forms of chl *a* in the PSI [37]. However, recent studies demonstrated that irradiating plants with a spectrum including FR in addition to VIS light leads to an increase in photosynthetic efficiency due to an enhancement of photochemical activity [38,39]. Moreover, by using a filter to remove photons above 700 nm from a solar spectrum (to quantify the effects on photosynthesis in diverse plant species), it was proved that FR photons (701 to 750 nm) are used for photosynthesis also by more complex photosynthetic organisms [40]. FR photons led to an increase in biomass productivity in Sun-adapted plants and especially in the leaves of shade-adapted ones, where they can be >50% of the total incident photons between 400 and 750 nm [40].

In light of these findings, we extended the investigation to eukaryotic OPOs that are phylogenetically distant from one another and inhabit different habitats. We utilized our recently developed experimental setup, which allows us to grow OPOs under selected non-terrestrial conditions [41]. The setup includes the Star Light Simulator, able to accurately generate different light intensities and spectra, including those of M-dwarf stars [19]. Among algal species, we selected two derived from primary endosymbiosis: *Chlorella vulgaris*, a freshwater green microalga, and *Dixoniella giordanoi*, a marine red unicellular microalga recently discovered and characterized [42,43]. Two microalgae derived from secondary endosymbiosis were also evaluated: *Microchloropsis gaditana*, a marine Eustigmatophyta, and *Chromera velia*, a marine chromeride with endosymbiotic capabilities. Among these species, one is not able to utilize FR (*C. vulgaris*), and one has demonstrated the ability to exploit it (*C. velia*) [32]. Instead, the ability to exploit FR of the two remaining species (*M. gaditana* and *D. giordanoi*) is not known yet. In addition to microalgae, we also tested two plants: *Physcomitrium patens* and *Arabidopsis thaliana*. *P. patens* is a moss representative of the Bryophytes, a group descendant from the early branching of plants upon land colonization [44] and a model organism for studies of physiology and plant evolution. *A. thaliana* is a flowering plant and the most utilized model organism in plant science, recently utilized also for astrobiological studies [45,46,47]. We performed a preliminary screening on the selected organisms, exposing them to a simulated M-dwarf light spectrum to test their survival, growth, and photosynthetic efficiency, comparing their responses to a simulated solar light spectrum and a far-red light.

## 2. Materials and Methods

### 2.1. Light Conditions

Three light spectra were utilized in this study: an M-dwarf (M7) simulated light spectrum, a solar (SOL) simulated light spectrum, and a far-red (FR) monochromatic light at 730 nm. A description of each light simulator employed and their respective emission spectra can be found in [19,20]. Light intensities were the following: SOL light had about 27.4 µmols of photons m^−2^ s^−1^ (5.6 W m^−2^) in the whole working range (380–780 nm), of which 20.3 µmols of photons m^−2^ s^−1^ (4.4 W m^−2^) in the PAR (400–700 nm); M7 had about 57.6 µmols of photons m^−2^ s^−1^ (10.2 W m^−2^) in the whole working range, of which 20.3 µmols of photons m^−2^ s^−1^ (4.3 W m^−2^) in the PAR; FR light had 30 µmols of photons m^−2^ s^−1^ (5 W m^−2^) in its working range (660–780 nm), of which 3 µmols of photons m^−2^ s^−1^ (0.3 W m^−2^) in the PAR. The PAR light emitted by the simulated M star is in the order of magnitude of that received by the very well-known Proxima Centauri b, an exoplanet orbiting the M-dwarf Proxima Centauri. Proxima Centauri b experiences on its surface irradiance of about 64–132 µmol photons m^−2^ s^−1^ (PAR) or about 3% that of the Earth [16].

### 2.2. Starting Biological Material

*Chlorella vulgaris* CCAP211-11B, *Microchloropsis gaditana* (formerly known as *Nannochloropsis gaditana*) CCAP849/5, *Chromera velia* CCAP1602/1, were all obtained from the Culture Collection of Algae and Protozoa (CCAP, SAMS Limited, Dunbeg, Scotland, UK); *Dixoniella giordanoi* instead is a recently discovered red alga [43] that was available in our laboratory. *M. gaditana*, *C. velia*, and *D. giordanoi* were maintained in F/2 medium [48], while *C. vulgaris* was maintained in BG-11 medium [49]. Cultures were maintained in the exponential phase of growth by renewing the cultures with fresh medium each week. During growth, all strains were exposed to the atmospheric air and kept in a climatic chamber (Piardi Tecnologie Del Freddo Srl, Brescia, Italy) at 28–30 °C under a continuous cool white, fluorescent light of 20 µmols of photons m^−2^ s^−1^ (L36W-840, OSRAM, Munich, Germany).

*Physcomitrium patens* (formerly known as *Physcomitrella patens*), Gransden wild type (WT) strain, was kindly provided by Prof. Alessandro Alboresi (University of Padua, Italy). Protonemal tissue of *P. patens* was maintained via vegetative propagation on solid PpNH_4_ medium [50] and grown in a climatic chamber (Criotecna, Altivole, Italy) at 24 °C, under a photoperiod composed of 16 h of white fluorescent light at 30 μmol photons m^−2^ s^−1^ (L36W-840, OSRAM, Munich, Germany) and 8 h of dark.

Regarding *A. thaliana*, seeds of the ecotype Columbia (Col-O) were kindly provided by Prof. Ildikò Szabò (University of Padua, Italy).

### 2.3. Experimental Plan

Each experiment was repeated twice at different times for every type of organism and relative treatment. A total of 6–8 biological replicates (6 for microalgae and *P. patens* and 8 for *A. thaliana*) were obtained.

For the acclimation experiments on microalgae, 20 µL of the selected strains from cultures kept in the exponential phase of growth were spotted on solid media depending on their species and requirements. The spots come from independent flasks. *C. vulgaris* was spotted on BG-11 + 1% agar plates at 3 optical densities at 750 nm (OD_750_): 1, 1.5, and 2. All other microalgal strains (*M. gaditana*, *C. velia*, and *D. giordanoi*) instead were spotted on F/2 + 1% agar plates at the same optical densities. Different plates were each exposed for 10 days to SOL, M7, and FR light at the light intensities reported above, at a constant temperature of 28–30 °C and terrestrial atmospheric composition and pressure (1 atm).

For the acclimation experiments on *P. patens*, 10-day-old plants grown in solid PpNO_3_ medium [50] and in the same environmental conditions previously reported for maintenance cultures were utilized. Growth tests started by spotting clones of 2 mm diameter on solid PpNO_3_ medium using a custom steel tubular stencil. The spots came from the same vegetatively-propagated culture but from different parts of the plate. Clones were grown for 7 days at room temperature in the same light conditions reported for microalgae.

For the acclimation experiments on *Arabidopsis thaliana*, seeds were sterilized and sowed in plates containing humid soil. Plates covered with clear plastic wrap were incubated for 3 days at 4 °C in the dark to allow stratification and synchronize germination. Plates were then transferred to a culture room (KK 1450 FIT P, POL-EKO, Wodzisław Śląski, Poland) with a photoperiod of 16 h of light/8 h darkness and light intensity of 80–100 μmol photons m^−2^ s^−1^ at 22 °C for 14 days to obtain seedlings with 2 rosette leaves. Seedlings were then picked, gently deprived of seeds, and transplanted into 7.5 cm diameter pots containing soil. To avoid soil dehydration during the experiments, approximately 2 cm of water around the pot’s base was maintained. Each pot containing about 8 seedlings has been exposed for 9 days to SOL, M7, and FR light in the respective growth chambers at room temperature, and at the same light conditions that were reported for microalgae and *P. patens.*

### 2.4. Growth Measurements

The growth of the microalgal strains in solid media was assessed at T_0_ = 0 days and T_f_ = 3 days via PAM imaging, using a FluorCam FC-800 (PSI, Photon Systems Instruments, Drasov, Czech Republic), by evaluating the ground chlorophyll fluorescence (F_0_) incremental ratio as described in [19]. To perform the F_0_ measurement, it was utilized only the measuring light, a faint pulsed red light (λ_max_ = 614 nm) at an intensity of 1.5 μmol of photons m^−2^ s^−1^, which allows measuring the fluorescence of the sample without enabling the photosynthetic process (non-actinic light). The other parameters set for the measurement were the shutter of the camera (set to 1) and its sensitivity (set to 22). After pre-adaptation in the dark for 30 min, samples were probed by this pulse-modulated light. Data obtained were then analyzed using the FluorCam7 software (PSI, Drasov, Czech Republic). The output of the analysis was an F_0_ value for each time point. F_0_ incremental ratios were obtained as follows:

(1) $$F_{0}\;\mathrm{Incremental}\;\mathrm{ratio}=\frac{\left[F_{0}\left(3\;d\right)-F_{0}\left(0\;d\right)\right]}{F_{0}\left(0\;d\right)}$$

where F_0_ (0 d) is the basal fluorescence at t = 0 days, and F_0_ (3 d) is the basal fluorescence at t = 3 days. We did not consider F_0_ data after 10 days because the F_0_ signal, utilizing the initial PAM setting, was saturating for most of the spots due to high cellular concentrations reached by some of the cultures. However, the phenotype of the samples was visually inspected.

After the treatment, plates containing *P. patens* spots were scanned in a Konica Minolta bizhub C280 (Tokyo, Japan) with a resolution of 600 ppi to quantify the growth as reported in [51]. In particular, it was calculated the integrated density (area × mean intensity) after the background exclusion with the ImageJ software (National Institutes of Health, Bethesda, MD, USA) “threshold colour” plugin. Integrated density was used to quantify instead of “area” because this parameter better takes into account the three-dimensional nature of *P. patens* colonies [52], the morphology of which is influenced by the different light regimes used for the growth.

*A. thaliana* growth was assessed by measuring the fresh weight of the entire 9-day-old seedlings after cleaning them from soil particles.

### 2.5. Photosynthetic Efficiency Measurements

The efficiency of the photosynthesis under M7 and SOL light spectra for the different organisms was evaluated using the F_v_/F_m_ parameter obtained via PAM imaging. To perform the F_v_/F_m_ measurement, in addition to the measuring light, a white light saturating pulse with λ_max_ = 569 nm was also utilized. To this aim, the “Super” parameter in the instrument was set to 50%, which corresponds to an intensity of the saturating impulse of about 2400 μmol of photons m^−2^ s^−1^. The shutter of the camera was set to 1 for all organisms, and its sensitivity was set to 22 for microalgae and 10 for *P. patens* and *A. thaliana*. The F_v_/F_m_ parameter, indicating PSII photosynthetic efficiency, was calculated as follows:

(2) $$\frac{F_{V}}{F_{m}}=\frac{\left[F_{m}\left(t\;f\right)-F_{0}\left(t\;f\right)\right]}{F_{m}\left(t\;f\right)}$$

where F_0_ (t f) is the basal fluorescence at the end of the experiments (3 days for microalgae, 7 days for *P. patens*, and 9 days for *A. thaliana*), and F_m_ (t f) is the maximum fluorescence at the end of the experiments, after 30 min of dark acclimation followed by a pulse of saturating light. F_v_/F_m_ parameter was not considered for organisms exposed to FR light since the F_v_ and F_0_ signals were too similar to obtain reliable results due to the absence of growth for most of them.

### 2.6. Statistical Analyses

Statistical analyses were performed utilizing Graph Pad Prism v9.5.1 software (Insight Partners, New York City, NY, USA). Data were calculated as the mean ± standard deviation of 6 (microalgae and *P. patens*) or 8 (*A. thaliana*) biological replicates and the comparison between different light conditions (M7, SOL, FR) for the same species was carried on with the one-way ANOVA technique (assuming the Gaussian distribution of data) followed by Tukey’s multiple comparison test (significance was set at *p* < 0.05).

## 3. Results

### 3.1. Microalgal Strains Grow and Photosynthesize Similarly in M7 and SOL

**Figure 1:**
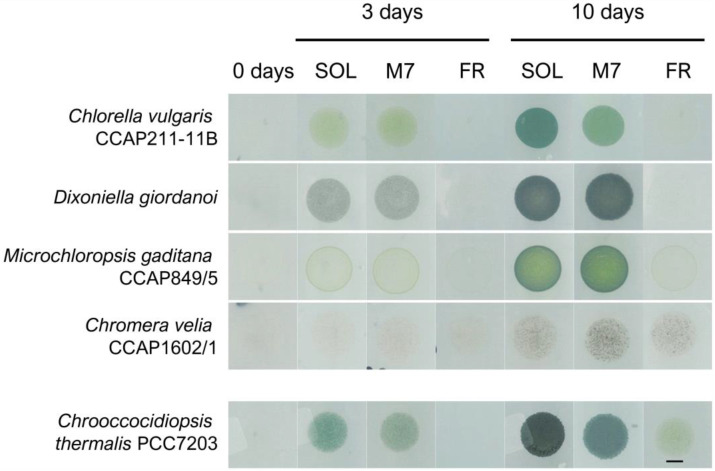
Example of phenotypes of the microalgal strains tested in the experiments after 0, 3, and 10 days under the three different light conditions. On the bottom row is reported the same kind of experiment for the cyanobacterium *Chrooccocidiopsis thermalis* PCC7203, previously reported in [19]. SOL, solar light; M7, M-dwarf light; FR, far-red light. Scale bar = 5 mm.

The exposure of the selected organisms to the three light conditions for 3 and 10 days yielded very different results (Figure 1).

The direct inspection of the Petri dishes after 3 and 10 days evidenced growth for all strains in SOL and M7 (Figure 1). All strains after 3 days and, more evidently, after 10 days had a higher cell density and were more pigmented under SOL and M7 with respect to day 0. For some strains, notably *C. vulgaris* and *C. velia*, differences in the pigmentation shown under SOL and M7 were found after 10 days. In FR, responses were more differentiated. *C. vulgaris* and *D. giordanoi* did not show any growth after 10 days with respect to day 0, while *C. velia* and *M. gaditana* appeared to be growing in this light condition.

**Figure 2:**
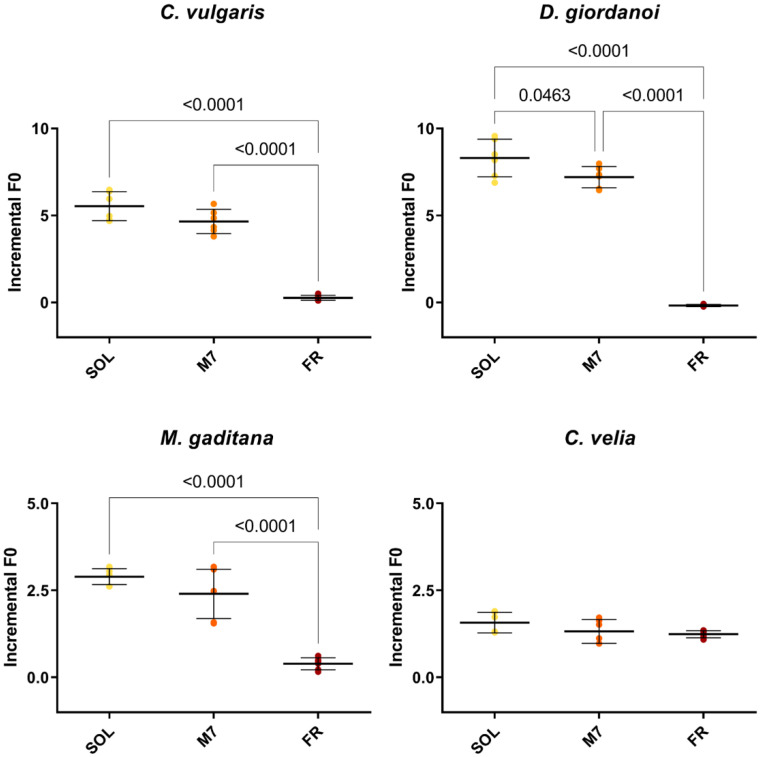
F_0_ incremental ratios after 3 days of the strains exposed to the different light conditions tested. The large bar shows the mean value of the data. The small bars show the standard deviation of the samples (6 biological replicates). *p*-values of the statistical analysis are reported on the graph if below the significance threshold (one-way ANOVA, significance was set at *p* < 0.05). SOL, solar light; M7, M-dwarf light; FR, far-red light.

Chlorophyll fluorescence measurements were made after 3 days to quantify the growth of the organisms (Figure 2) and their photosynthetic efficiency (Table 1). All strains showed similar levels of F_0_ incremental ratios under SOL and M7 but different absolute values. Under FR, almost all strains showed very low levels of F_0_ incremental ratios compared to SOL and M7. Surprising was the result of *C. velia,* which showed similar values of F_0_ incremental ratio under all light conditions tested. The photosynthetic efficiency of the strains was then evaluated for M7 and SOL lights. All strains maintained similar values of F_v_/F_m_ in M7 and SOL conditions, except for *C. velia*, which showed a higher F_v_/F_m_ in M7 with respect to SOL.

### 3.2. Physcomitrium patens Shows Reduced Growth in M7 but Has Normal Development and Photosynthetic Efficiency

**Figure 3:**
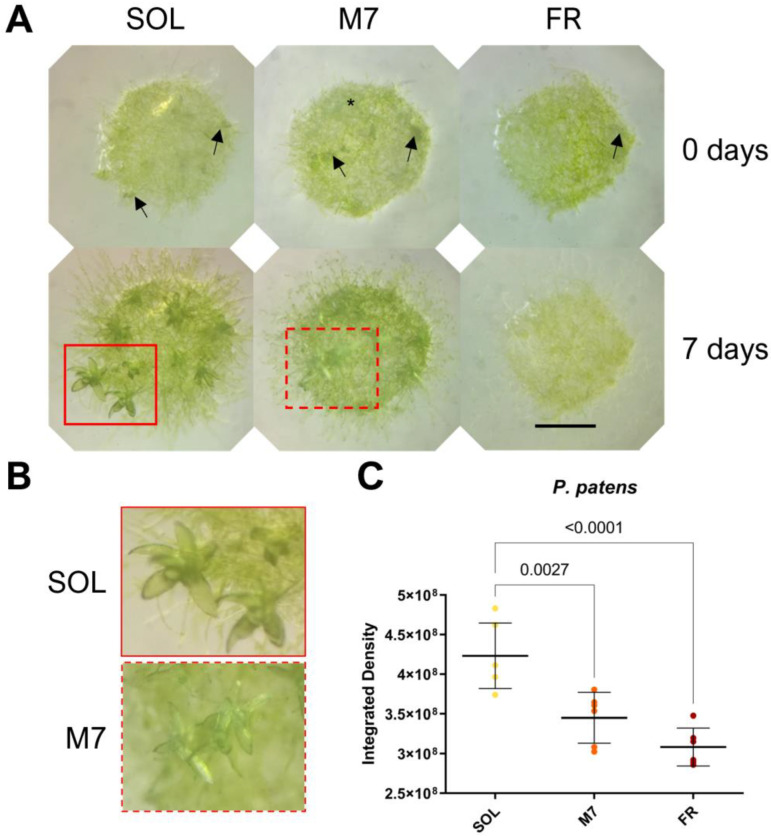
*P. patens* 7 days acclimation experiment. (**A**) Images of *P. patens* spots at 0 and 7 days under the three different light conditions; black arrows indicate gametophores buds, red rectangles indicate gametophores, and the black asterisk indicates protonema; (**B**) close up of gametophores in SOL and M7 after 7 days (in FR no development was observed); (**C**) growth of *P. patens* under the three different light conditions. The large bar shows the mean value of the data. The small bars show the standard deviation of the samples (6 biological replicates). *p*-values of the statistical analysis are reported on the graph if below the significance threshold (one-way ANOVA, significance was set at *p* < 0.05). SOL, solar light; M7, M-dwarf light; FR, far-red light. Scale bar = 1 mm.

The acclimation and growth of *P. patens* were followed for 7 days under the selected light conditions (Figure 3).

Visual inspection of *P. patens* spots (Figure 3A,B) showed that plants survived in all conditions tested. After 7 days, both SOL- and M7-acclimated plants developed the protonema (the earliest stage of development of the gametophyte in mosses) and existing gametophores (where gamete production occurs) and started to develop new buds (from which gametophores will form). The lateral development of protonema appeared rarer in M7 with respect to SOL, but M7-acclimated samples overall did not show evident growth defects. FR-acclimated plants after 7 days overall did not grow. After 7 days, they appeared yellowish in color with respect to the other light conditions; moreover, they developed a protonema but did not show any evident development of existing gametophores or the formation of new buds. The quantification of growth using the integrated density of the spots (Figure 3C) showed that SOL-acclimated plants yielded the highest growth, followed by M7- and FR-acclimated plants. Chlorophyll fluorescence measurements after 7 days (Table 2) showed that photosynthetic efficiency remained similar for SOL and M7-acclimated samples.

### 3.3. Arabidopsis thaliana rows under M7 but Shows a Shade-Avoidance Response

**Figure 4:**
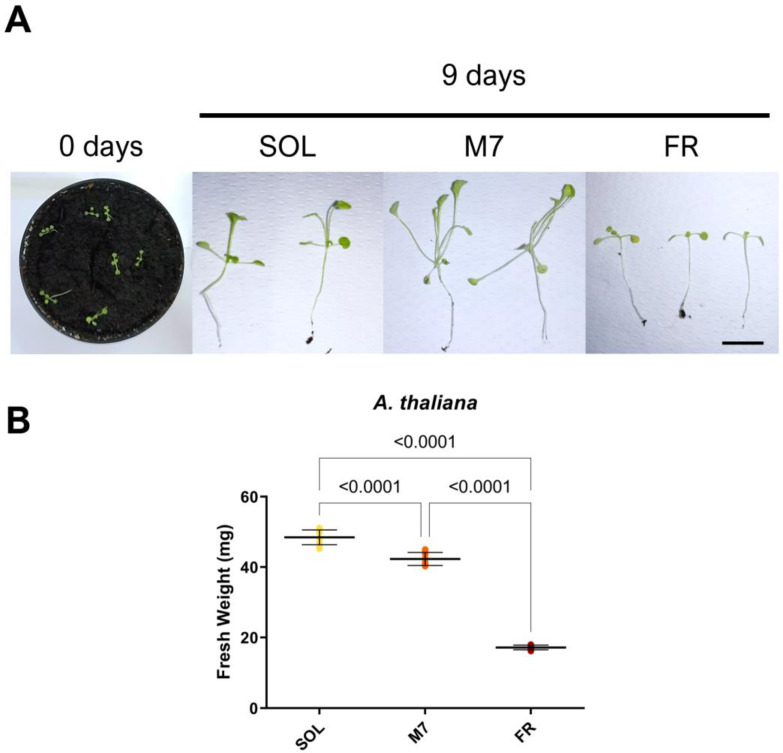
*A. thaliana* seedlings acclimation experiment. (**A**) Images of *A. thaliana* seedlings at 0 and after 9 days under the three different light conditions; (**B**) Fresh weight of *A. thaliana* seedlings at the end of the experiment under the three different light conditions. The large bar shows the mean value of the data. The small bars show the standard deviation of the samples (8 biological replicates). *p*-values of the statistical analysis are reported on the graph if below the significance threshold (one-way ANOVA, significance was set at *p* < 0.05). SOL, solar light; M7, M-dwarf light; FR, far-red light. Scale bar = 10 mm.

Finally, the acclimation of *A. thaliana* seedlings was investigated after 9 days in the three light conditions (Figure 4).

*A. thaliana* seedlings survived under all light conditions, as evidenced by visual inspection (Figure 4A). At the tested light intensities, SOL-acclimated seedlings showed elongated hypocotyls and petioles. M7-acclimated seedlings had a more pronounced phenotype with respect to SOL-acclimated ones, with hyponastic (upward-oriented) and yellowish leaves, besides elongated hypocotyls and petioles. FR-acclimated seedlings instead showed an arrest in development. The biomass of the seedlings was then quantified (Figure 4B). SOL-acclimated seedlings showed the highest biomass, followed by M7-acclimated seedlings and, finally, FR-acclimated seedlings. Fluorescence measurements after 9 days (Table 3) again showed no differences between the photosynthetic efficiency of SOL and M7-acclimated samples.

## 4. Discussion

Oxygenic photosynthetic organisms have high attention as targets for the search for life outside the Solar System. They are able to generate surface biosignatures such as the vegetative red edge (VRE), Earth’s spectral feature related to the reflectance properties of photosynthetic pigments in visible and infrared lights [53]. They also generate atmospheric biosignatures, such as oxygen and its photoproduct ozone, derived from oxygenic photosynthesis [9]. Both biosignatures can be detected via remote sensing spectroscopy [10,54]. Much was theorized about the possibility of oxygenic photosynthesis [5,10,12,16,17,18,55] around exoplanets orbiting the HZ of M-dwarfs, but so far, few experimental data about the survival and photosynthetic efficiency of OPOs to M-dwarf light spectra have been generated [19,20]. These experiments were performed using the most simple and adaptable oxygenic photosynthetic organisms on Earth, cyanobacteria. Here, we focused our attention on more complex forms of OPOs, microalgae, and plants, performing a preliminary study on their survival, growth, and photosynthetic efficiency.

Regarding the acclimation of microalgae, all strains grew similarly under SOL and M7, even if at growth rates that were species specific. Similar growths in SOL and M7 for strains not able to utilize FR alone were not expected but can be explained. *C. vulgaris* has been successfully cultivated in white light with added FR light in the past [56,57], even if the growth was higher in the VIS-dominated spectrum than in the FR-dominated one. The responses seen in our experiments for all microalgae in M7 could rely upon the Emerson enhancement effect [58], as recently demonstrated for horticultural and agronomical plants [39,40,59]. When provided together with VIS light (as in the M7 spectrum), FR photons have a synergistic effect; they preferentially excite PSI, contributing to even the excitement of the two photosystems working in series, PSII and PSI, so that the photochemistry can pursue optimality and the photosynthetic rate can be boosted. Even though this was acknowledged more than 60 years ago, it was not until recent times that the scientific community started to consider the potentiality of FR light in photosynthesis. Lately, it has been, thus, proposed to widen the concept of PAR to “extended PAR” (ePAR), which would comprehend wavelengths up to 750 nm [47]. Instead, under FR only, the growth of every microalgal strain tested was very low, except for *C. velia*. *C. velia* is an endosymbiont of the coral species *Plesiastrea versipora* and *Leptastrea purpurea* [60], meaning it lives inside their tissues. This alga can, moreover, switch between a VIS and an FR-shifted light-harvesting antenna system upon illumination with blue or FR light, respectively [61]. In this way, *C. velia* can photosynthesize in these microniches (inside the coral’s tissues), characterized by stronger components of FR light but very low irradiance in the VIS due to the depletion of light by other photosynthetic symbionts such as dinoflagellates [62]. This adaptation allowed the strain to acclimate and grow in FR, but possibly also in M7, a spectrum of light that is surprisingly similar to that experienced by the organism in its natural environment [62]. Moreover, of interest was the apparent growth of *M*. *gaditana* after 10 days under the FR light. *Microchloropsis* genus indeed has never been reported to use FR light [32]. The species, however, is phylogenetically close to a recently discovered species, an Eustigmatophyte called FP5, able to grow under FR only [35]. *M. gaditana* might be exhibiting some kind of yet unknown FR acclimation or might be using the little portion of VIS light in the FR spectrum to grow (a shoulder of emission of about 3 µmol of photons m^−2^ s^−1^ in the range 680–700 nm). This present study could not investigate the pigment content and photosystem organization of the strain acclimated to the different light conditions; therefore, further investigation will be needed to address this peculiarity. Finally, some novel information on the growth capabilities under different spectra of the newly isolated *D. giordanoi* was obtained. *D. giordanoi* is a unicellular rhodophyte (red alga) recently discovered [43] and for which the photosynthetic apparatus and acclimation to three light intensities were characterized [42]. *D. giordanoi* was shown to acclimate, modulate its chlorophyll and phycobiliprotein content, and re-organize thylakoidal membranes in response to different light intensities of a white LED lamp. However, the acclimation properties of the strain under different light spectra were not tested so far. Our experiments showed *D. giordanoi* can survive and grow similarly in SOL and M7 light regimes but is unable to grow in FR alone. The inability of this alga to utilize FR is in line with the current knowledge. There are very few microalgae known to utilize FR for photosynthesis, and none of them belongs to the phylum Rhodophyta. Among FR-utilizing algae, inside the green lineage, there is the microalga *Ostreobium* sp. [63], while outside the green lineage, there are several algae derived from secondary or tertiary endosymbiotic events and generally classified into the supergroup Stramenopila-Alveolata-Rhizaria (SAR): an alveolate, *Chromera velia* [34], 2 eustigmatophytes, FP5 and *Trachydiscus minutus* [33,35], and the diatom *Phaeodactylum tricornutum* [64]. The responses of microalgae appear similar to those observed for cyanobacteria able or unable of FaRLiP response [19]. In that work, cyanobacterial strains tested were able to survive and utilize the simulated M-dwarf spectrum to grow and photosynthesize efficiently. Moreover, we also demonstrated that different species employed different acclimation strategies, which anyhow led to efficient oxygen production [20]. Their ability to utilize the simulated M-dwarf spectrum could reflect the habitat these microorganisms occupy on Earth. Light can be quantitatively and qualitatively different in aquatic environments or terrestrial ones [65]. Light penetration in water, especially in the PAR (400–700 nm) and UVA (315–400 nm) wavebands, is attenuated more easily with respect to air through particulate but also via the absorption of photosynthetic microorganisms and water plants [66]. Most aquatic photosynthetic organisms, therefore, are adapted to shade [67] and possess an inventory of pigments to match from time to time at different depths the spectral characteristics of the light available [66]. These adaptations, together with the Emerson enhancement effect, could explain why microalgae and cyanobacteria could acclimate well to M7 and grow similarly to SOL.

By increasing the complexity of the organisms though, responses start to change. *P. patens* results are in line with those previously found for the organism in red and FR light [68,69]. Under FR light, there was no bud development and, therefore, no gametophore formation. Our results, however, showed that M7-acclimated plants had a phenotype that was more similar to SOL-acclimated samples than to FR-acclimated ones. Gametophores developed but slowly. This is in line with the finding that under red light, the development of protonema and gametophores is delayed with respect to the growth under white light [68]. M7 light is reddish compared to SOL light, and this might explain the similar results obtained. Regarding *A. thaliana*, seedlings exposed to SOL grew but showed signs of a low-light response, including elongated hypocotyls and petioles, as the result of the low light intensity utilized, suboptimal for the growth of this species. Seedlings exposed to M7 also grew, showing a peculiar light-induced response. In heliophyte flowering plants, such as *A. thaliana*, a red-to-FR imbalance, typical of the light spectrum received under a canopy, triggers morphophysiological responses described as shade-avoidance syndrome. This includes the elongation of petioles and the rise of the leaf tips, similar to low VIS light [70], a change in the ratio between the photosystems and their antenna size, and strong photoinhibition upon re-exposure to direct sunlight [71]. Seedlings in M7 needed higher light intensities to grow normally and suffered from the low ratio between red and FR light in the M7 spectrum, which triggered the shade-avoidance syndrome. This is in line with what is seen for this organism under low red/FR ratios [72] and under monochromatic FR light [71]. In the latter study, Hu and collaborators [71] showed the acclimation of *A. thaliana* to FR light. In this light condition, after 7 days, they observed plants with elongated, paler petioles and hyponastic and yellowish leaves. In our experiment, M7-acclimated seedlings showed indeed a similar phenotype to their FR-acclimated plants. Instead, our seedlings in FR did not grow, and we could not observe morphophysiological features. These differences in the responses can be explained through the different cultivation strategies employed. For their experiment, they opted for cultivating the plants for 5 weeks in white light (total light intensity 120 µmols m^2^ s^−1^, 12 h/12 h day/night cycle) before switching to FR light (total light intensity 100 µmols m^2^ s^−1^, same day/night cycles) for 7 days. This means that they tested the acclimation of adult plants to FR. In our experiment, instead, we cultivated seedlings for 9 days in continuous FR light at a much lower intensity (30 µmols m^2^ s^−1^). The scarcity and the quality of photons utilizable for photosynthesis led then to the inhibition of growth in FR.

A major adaptation for land colonization was the use of a different light spectrum. On land, photosynthetic organisms gained access to the full spectrum of the sunlight (just attenuated by the atmosphere). Thus, they progressively had to adapt to a different spectral distribution of the photons and the inevitable high irradiances [73,74]. In mosses, this adaptation was not fully in place yet, also due to the peculiar niches they inhabit. A biochemical and structural study [75] on *P. patens* recently revealed the presence of a PSI antenna that facilitates the adaptation of this moss to low-light conditions, in contrast to the more complex flowering plants, such as *A. thaliana*, which have a different PSI antenna, adapted to high-light conditions. This feature could explain the different responses seen in our experiments. *P. patens*, capable of acclimating to low light, can have a normal development under M7, even if its growth is slower when compared to SOL, given the different spectral distribution of the photons available. *A. thaliana*, adapted to high light, in addition to experiencing a different spectral distribution in M7, is also energy-limited. Therefore, it cannot develop normally, showing signs of shade-avoidance syndrome.

Exotic adaptations have been often invoked for organisms exposed to an M-dwarf spectrum: oxygenic photosynthesis utilizing infrared wavelengths, using multiple chained photosystems, more photons per molecule of oxygen, or non-chlorophyll, FR-absorbing pigments [10,13,18,76]. Taken together, however, our results support the hypothesis that exotic adaptations are not required. VIS light would be enough for oxygenic photosynthesis under an M-dwarf spectrum as theoretical works estimated, albeit with lower global productivity than on Earth [5,16,17]. Growth and photosynthetic efficiency data suggest that the majority of oxygenic photosynthetic organisms could utilize an M-dwarf light spectrum to photosynthesize and grow. Organisms similar to terrestrial sciophytes (evolved under low and VIS-filtered light) would be probably advantaged under this light spectrum, while organisms similar to terrestrial heliophytes (evolved under full sunlight) would be disadvantaged by the quantity and quality of light emitted by the star. This preliminary work suggests that the evaluation of the photosynthetic responses of sciophyte and heliophyte higher plants will help to constrain which kind of biospheres we could expect from exoplanets orbiting M-dwarf stars.

## 5. Conclusions

After the discovery of thousands of exoplanets orbiting their parent stars, finding life beyond the Solar System could be one of the most important steps in mankind. Terrestrial-like exoplanets orbiting the Habitable Zone of M-dwarfs could be good places where to start. Their distances from us, however, require probing the presence of life through remote sensing and biosignatures, i.e., signs of life. Oxygenic photosynthetic organisms produce gaseous and surface biosignatures, arising from the molecular oxygen produced during photosynthesis and from the reflectance properties of their photosynthetic pigments, respectively. To generate biosignatures, organisms first would need to harvest the light from the M-dwarf star. Results of this work show that the light of a simulated M-dwarf spectrum can be harvested, even efficiently, by terrestrial photosynthetic pigments. Together with previously published results on cyanobacteria [19,20], they lead us to reconsider the possibility and capability of the growth of oxygenic photosynthetic organisms, complex ones included, on planets orbiting M-dwarf stars. Microalgae, being well adapted to changes in light quality and quantity, do not suffer this simulated light regime (M7) and grow normally. *P. patens* is also adapted to low light and develops normally, but compared to microalgae is more complex, requires more energy, and cannot take full advantage of the spectral distribution of the photons in M7, thus growing more slowly under this light condition than in solar light (SOL). *A. thaliana* instead is not adapted to M7, due to both the different spectral distribution and the low intensity, and shows, therefore, an altered development, even if it can utilize the simulated light to photosynthesize. These results show that oxygenic photosynthetic organisms could harvest the M-dwarf light with photosynthetic pigments not necessarily different from terrestrial ones. Simpler OPOs would be probably advantaged under an M-dwarf light regime, more complex OPOs would be, instead, disadvantaged by the different spectral distribution and the low light. Anyhow, OPOs living in these conditions could lead to the generation of surface biosignatures based on chlorophyll *a* and could generate gaseous biosignatures based on O_2_ from photosynthesis. Such findings are promising in the frame of finding signs of oxygenic life arising from the atmospheres and the surfaces of these distant worlds by present and future space missions.

## Figures and Tables

**Table 1:** Photosynthetic efficiency (F_v_/F_m_) after 3 days of the strains in SOL and M7. Data are presented as averages ± standard deviation of six biological replicates (N = 6). Different letters highlight significant differences between light conditions within the same strain. (one-way ANOVA, *p*-value < 0.001). SOL, solar light; M7, M-dwarf light.

Organism	F_v_/F_m_	
	SOL	M7
*C. vulgaris*	0.61 ± 0.02 ^a^	0.58 ± 0.05 ^a^
*D. giordanoi*	0.55 ± 0.01 ^a^	0.55 ± 0.01 ^a^
*M. gaditana*	0.69 ± 0.01 ^a^	0.69 ± 0.01 ^a^
*C. velia*	0.40 ± 0.02 ^a^	0.45 ± 0.02 ^b^

**Table 2:** Photosynthetic efficiency (F_v_/F_m_) of *P. patens* after 7 days in SOL and M7. Data are presented as averages ± standard deviation of 6 biological replicates. Different letters highlight significant differences between light conditions within the same strain. (For one-way ANOVA, significance was set at *p*-value < 0.05.) SOL, solar light; M7, M-dwarf light.

Organism	F_v_/F_m_	
	SOL	M7
*P. patens*	0.79 ± 0.02 ^a^	0.78 ± 0.04 ^a^

**Table 3:** Photosynthetic efficiency (F_v_/F_m_) of *A. thaliana* after 9 days in SOL and M7. Data are presented as averages ± standard deviation of 8 biological replicates. Different letters highlight significant differences between light conditions within the same strain. (For one-way ANOVA, significance was set at *p*-value < 0.05.) SOL, solar light; M7, M-dwarf light.

Organism	F_v_/F_m_	
	SOL	M7
*A. thaliana*	0.77 ± 0.03 ^a^	0.75 ± 0.02 ^a^

## Data Availability

Data are contained within this article and Supplementary Materials.

## Supplementary Materials

The following supporting information can be downloaded at: https://www.mdpi.com/article/10.3390/life13081641/s1. Figure S1: Images of *P. patens* spots at 0 and 7 days under the three different light conditions.
