# Supplementary material for: Growth and Photosynthetic Efficiency of Microalgae and Plants with Different Levels of Complexity Exposed to a Simulated M-Dwarf Starlight
Source: Life (Basel). 2023 Jul 28;13(8):1641. doi: 10.3390/life13081641 (PMC10455698; doi:10.3390/life13081641)
Supplement: Supplementary file 1 [file life-13-01641-s001.zip › life-2477054-supplementary.pdf]

life

Article

Supplementary Material for: Growth and Photosynthetic Efficiency of Microalgae and Plants with Different Levels of Complexity Exposed to a Simulated M-Dwarf Starlight

Mariano Battistuzzi [1,2,3,*], Lorenzo Cocola [1], Elisabetta Liistro [2], Riccardo Claudi [4,5], Luca Poletto [1] and Nicoletta La Rocca [2,3]

1 National Council of Research of Italy, Institute for Photonics and Nanotechnologies (CNR-IFN), 35131 Padua, Italy; lorenzo.cocola@cnr.it (L.C.)
2 Department of Biology, University of Padua, 35121 Padua, Italy; nicoletta.larocca@unipd.it (N.L.R.)
3 Center for Space Studies and Activities (CISAS), University of Padua, 35131 Padua, Italy
4 National Institute for Astrophysics (INAF), Astronomical Observatory of Padua, 35122 Padua, Italy; riccardo.claudi@inaf.it
5 Department of Mathematics and Physics, University Roma Tre, 00146 Rome, Italy
* Correspondence: mariano.battistuzzi@pd.ifn.cnr.it

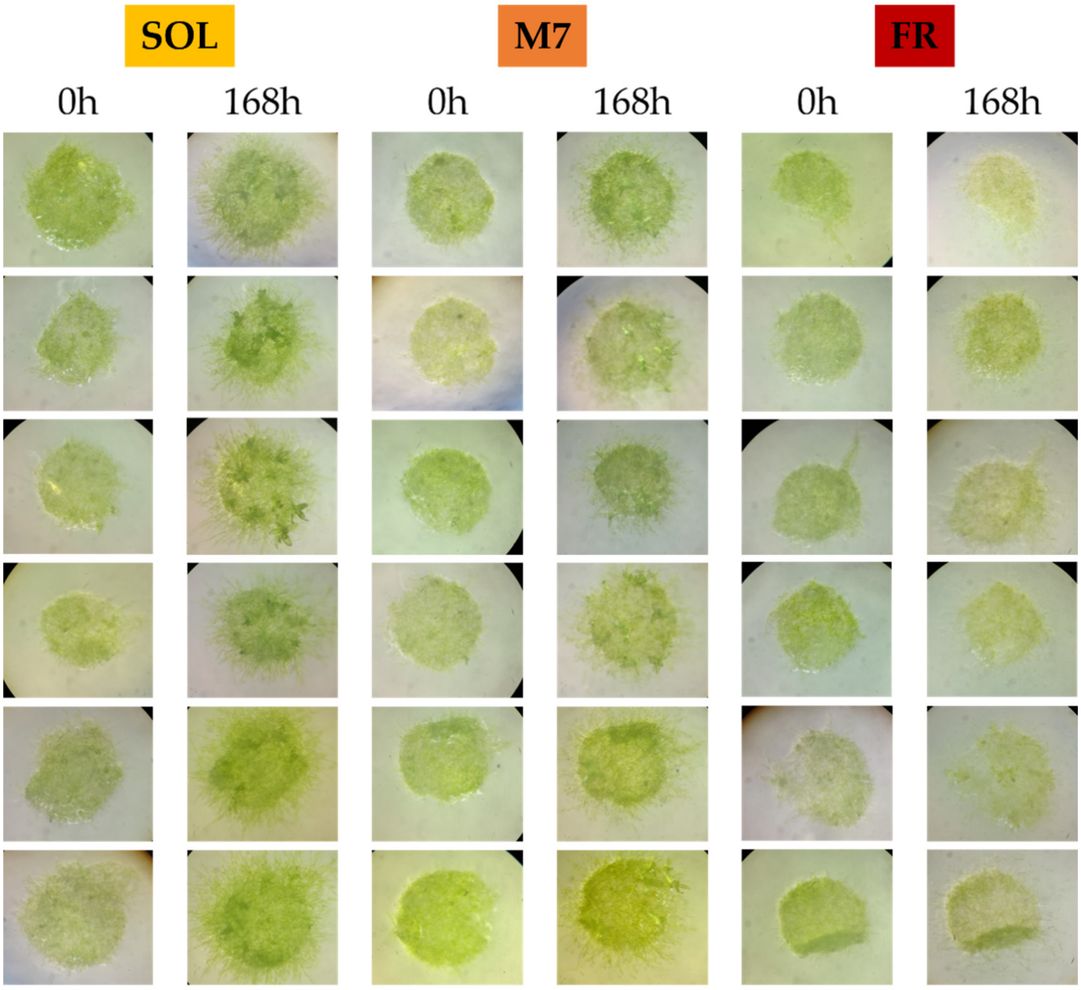

Figure S1. Images of *P. patens* spots at 0 and 7 days under the three different light conditions. SOL, Solar light; M7, M-dwarf light; FR, Far-red light.
